# Comparison of parameters derived from a three-minute all-out test with classical benchmarks for running exercise

**DOI:** 10.1371/journal.pone.0266012

**Published:** 2022-03-24

**Authors:** Filipe A. B. Sousa, Fúlvia B. Manchado-Gobatto, Natália A. Rodrigues, Claudio A. Gobatto

**Affiliations:** 1 Laboratory of Applied Sport Physiology, University of Campinas, Campinas, São Paulo, Brazil; 2 Post-Graduation Program in Nutrition, Institute of Physical Education and Sport, Federal University of Alagoas, Maceio, Alagoas, Brazil; University of Calgary, CANADA

## Abstract

This study aimed to compare four constructs from the three-minute all-out test (AO3)–end power (EP), the area above EP (WEP), maximum power (Pmax), and attained $\dot{V}O_{2\mathrm{peak}}$−to those derived from the classical CP model in tethered running. Seventeen male recreational runners underwent two experiments to test for reliability and agreement of AO3 parameters with those obtained from the classical CP model (Wꞌ and CP), a graded exercise test ($\dot{V}O_{2\max}$) and a 30-second all-out test (AO30s; Pmax); all performed on a non-motorized treadmill (NMT). Significance levels were set at p<0.05. There were no significant differences between test-retest for Pmax (p = 0.51), WEP (p = 0.39), and EP (p = 0.64), showing generally close to zero bias. Further, retest ICC were high for Pmax and EP (ICC > 0.86) but moderate for WEP (ICC = 0.69). Pmax showed no difference between AO3 and AO30s (p = 0.18; CV% = 9.5%). EP and WEP disagreed largely with their classical critical power model counterparts (p = 0.05; CV%>32.7% and p = 0.23; CV%>39.7%, respectively), showing greater error than their test-retest reliability. $\dot{V}O_{2\mathrm{peak}}$ from AO3 was not different (p = 0.13) and well related (CV% = 8.4; ICC = 0.87) to the incremental test $\dot{V}O_{2\max}$. Under the studied conditions, the agreement of EP and WEP to CP and Wꞌ was not strong enough to assure their use interchangeably. Pmax and $\dot{V}O_{2\max}$ were closer to their criterion parameters.

## 1. Introduction

Relying heavily on the bioenergetic basis of the traditional critical power model, an all-out test lasting three minutes (AO3) has been described in the late 2000s as an advantageous alternative to determine the critical power (CP) on a cycle ergometer [1–3]. The proposition of AO3 stems from its ability to predict the power-duration relationship in a single test session. On the other hand, the traditional CP model needs three to four constant-work-rate efforts (CWR) until exhaustion. This more established protocol is based on its ability to reveal the highest sustainable work rate for a prolonged period of time–for example, ranging 20.5 to 67.4 min for cycle ergometry according to the findings of Black et al. [4]–or the critical power (CP), and its curvature constant (Wꞌ), which is the finite total amount of work one can perform above CP [5]. Linear formulations of this relationship can be performed by plotting power output (P) against the inverse of time to exhaustion (t):

$$P=\frac{W\prime }{t}+CP$$ (Eq 1)

Considering Eq 1, when Wꞌ is completely depleted, P equals CP. So, one sufficiently long all-out effort would be an interesting alternative to obtain CP based on the test’s end power (EP), while the area above EP (WEP) could serve as an estimate of Wꞌ [2]. Early data on the AO3 performed on a cycle ergometer had shown test-retest reliability for end power (3% of CV; ICC at 0.99) [1] and close agreement between CP and EP (6 W of typical error; Pearson’s r = 0.99). Further, despite limitations that have been reported when comparing Wꞌ and WEP (2.8 kJ of typical error; Pearson’s r = 0.84) [2], both are often considered equivalent and valuable for practical applications [6, 7].

Data following the first investigations of AO3 presented controversial results about its parameters compared to the established criterion. It is not unusual to find experimental data indicating proper reliability of AO3 parameters [8, 9]. However, EP level of concordance to the CWR-determined CP may be corroborated or challenged (8–11). This trend is similar for activities other than cycling, such as swimming [10–13], rowing [14], arm crank cycle [15], isokinetic cycle [16, 17], and running [18–20], with varied results on the agreement.

For the non-sustainable work capacity, there is evidence associating WEP to the Wꞌ obtained from the CWR model and muscle metabolic responses from high-intensity exercise [21]. However, this can also be disputed [9, 22, 23]. The agreement between WEP determined by AO3 and Wꞌ by CWR may depend on the mathematical fitting, being higher when Wꞌ is determined by the linear models [24].

Arguments can be made about the peak rate of oxygen consumption ($\dot{V}O_{2\mathrm{peak}}$) determined after an AO3 [1], as well as the maximum mechanical power attained. The former has already been tested against the verified maximum rate of oxygen consumption ($\dot{V}O_{2\max}$) attained during a graded exercise test on the cycle ergometer, showing both positive [1, 21, 25] and negative agreement [8, 18, 26, 27]. Regarding maximum mechanical power, tests typically limited to 90 s have been used for this purpose [28, 29]. However, a comparison of the maximum mechanical power (Pmax) obtained during the AO3 to that from a shorter duration test is yet to be tested.

As most of the evidence on AO3 parameters is based on a cycle ergometer, the protocol’s immediate use may be troubling for running. Propositions often use velocity instead of power to control for the external load [18–20], and at least in a shuttle running scenario [30, 31], the need for more precise control of work-rate has been suggested [32]. An interesting way that may overcome this issue is the use of tethered running on a non-motorized treadmill (NMT), which may provide reliable [33] and partially valid [34] AO3 parameters controlled by mechanical power, in addition to a continuous measurement of accelerated running in a controlled environment.

This study aimed to test the agreement of the four AO3 constructs to classically derived parameters, all of them performed in a unique setup for tethered running. Specifically, the study was divided into two phases: i) to test for reliability and agreement of AO3 parameters with a 30-second all-out test; and ii) compare AO3 parameters with the ones obtained from the classical CP model (Wꞌ and CP) and a graded exercise test ($\dot{V}O_{2\max}$); all performed within five test sessions on an NMT. The experimental hypothesis is that EP, WEP, Pmax, and $\dot{V}O_{2\mathrm{peak}}$ obtained during the tethered running AO3 would present good reliability and high agreement to CP and Wꞌ from the classical critical power model Pmax from a shorter all-out effort, and $\dot{V}O_{2\max}$ from the graded exercise test.

## 2. Materials and methods

### 2.1. Experimental design

**Fig 1:**
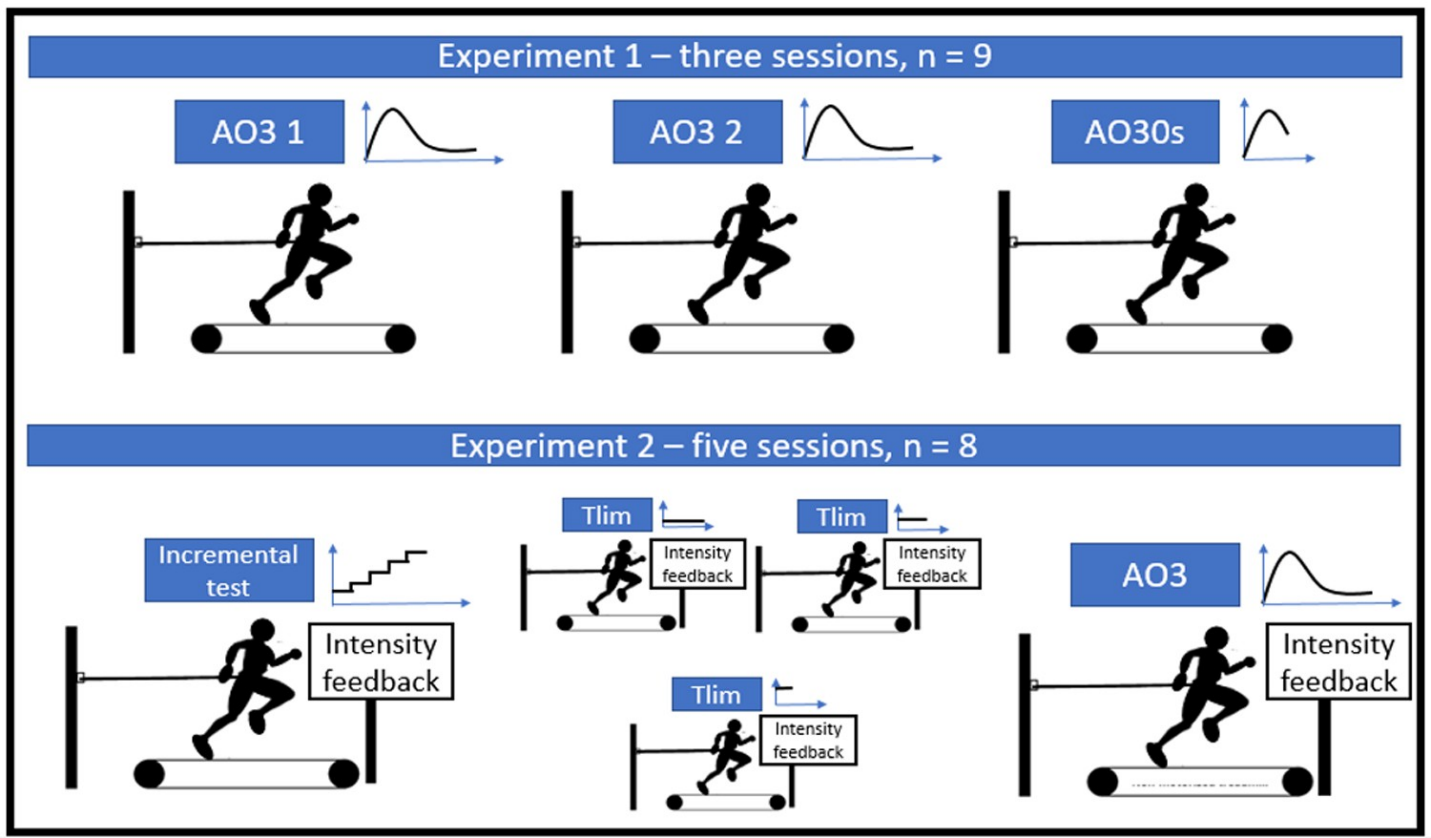
The study design. Study’s phase 1 tested the reliability of AO3 parameters and Pmax comparison to AO30s. Study’s phase 2 had a graded exercise test, three CWR intensities in a randomized order, finishing with one AO3. CWR was used for the classical CP model. All sessions were performed with 24–72 h intervals between them.

The study was divided into two phases (Fig 1), both in a repeated measure, single-blinded, crossover design. One of the phases was set to determine the AO3 parameter’s reliability and comparison to Pmax (n = 9), while the second phase intended to verify the level of agreement of AO3 obtained parameters (WEP, EP, and $\dot{V}O_{2\mathrm{peak}}$) compared to the classical critical power model and other physiological benchmarks (n = 8). Volunteers underwent ergometer familiarization before each of the study’s phases, running at will and performing short sprints. In the first phase, volunteers underwent three test sessions. In two of them, each volunteer performed an AO3; the results were then compared to measure reliability. In the last test session, a 30 s all-out effort was performed for comparison to the maximum power output obtained during the AO3.

For the second phase of the study, volunteers underwent five test sessions. In the first, a graded exercise test was performed. The next three comprised one constant-work-rate test (CWR) until exhaustion, under different work rates and in random order; the fifth, they performed an AO3 test. This second phase allows for determination of CP and Wꞌ from the classical critical power model and $\dot{V}O_{2\max}$ from the graded exercise test to compare with the AO3 parameters. All tests were conducted on an instrumented non-motorized treadmill to measure resultant force and velocity.

### 2.2. Volunteers

Seventeen male recreational runners gave their voluntary consent to participate in this study (age = 26 ± 9 years; height = 174 ± 5 cm; weight = 67.7 ± 12.4 kg; body fat = 8.5 ± 4.4%; training frequency ≥ 4 days/week; volume ≥ 15 km/week). During test procedures, volunteers were encouraged to maintain the same food intake and hydration habits and reduce training intensity 24 h before test sessions. All individuals were informed of test procedures and gave written consent to voluntarily participate in this investigation, which complied with the declaration of Helsinki. The Research Ethics Committee of the School of Medical Sciences approved all procedures (CAAE no. 28442314.0.0000.5404).

### 2.3. Procedures

Volunteers performed a 10-minute constant work-rate warm-up on the non-motorized treadmill for all test sessions. The external load was regulated by online visual feedback, given by a large monitor (42 inches) placed directly in front of the treadmill. The monitor displayed power performed in the horizontal plane. Before testing, the warm-up work rate was below individual LT, around 100 W. Ten minutes of passive rest was given between warm-up and exercise.

The feedback for work rate was removed when the all-out efforts were performed, and no information about test time to completion was provided. Strong verbal encouragement by the same researchers was given to the volunteers to consistently perform at their maximum intensity (33,34). Resultant power from AO3 was smoothed using a rolling average filter (5 s window). The maximum mechanical output was obtained as the highest value averaged in any given 1 s interval [35]. For the AO3, EP was calculated as the average of resultant power in the last 30 seconds, while WEP was the area above EP from the power vs. time curve (33). All mechanical data was presented in units relative to the body mass.

**Fig 2:**
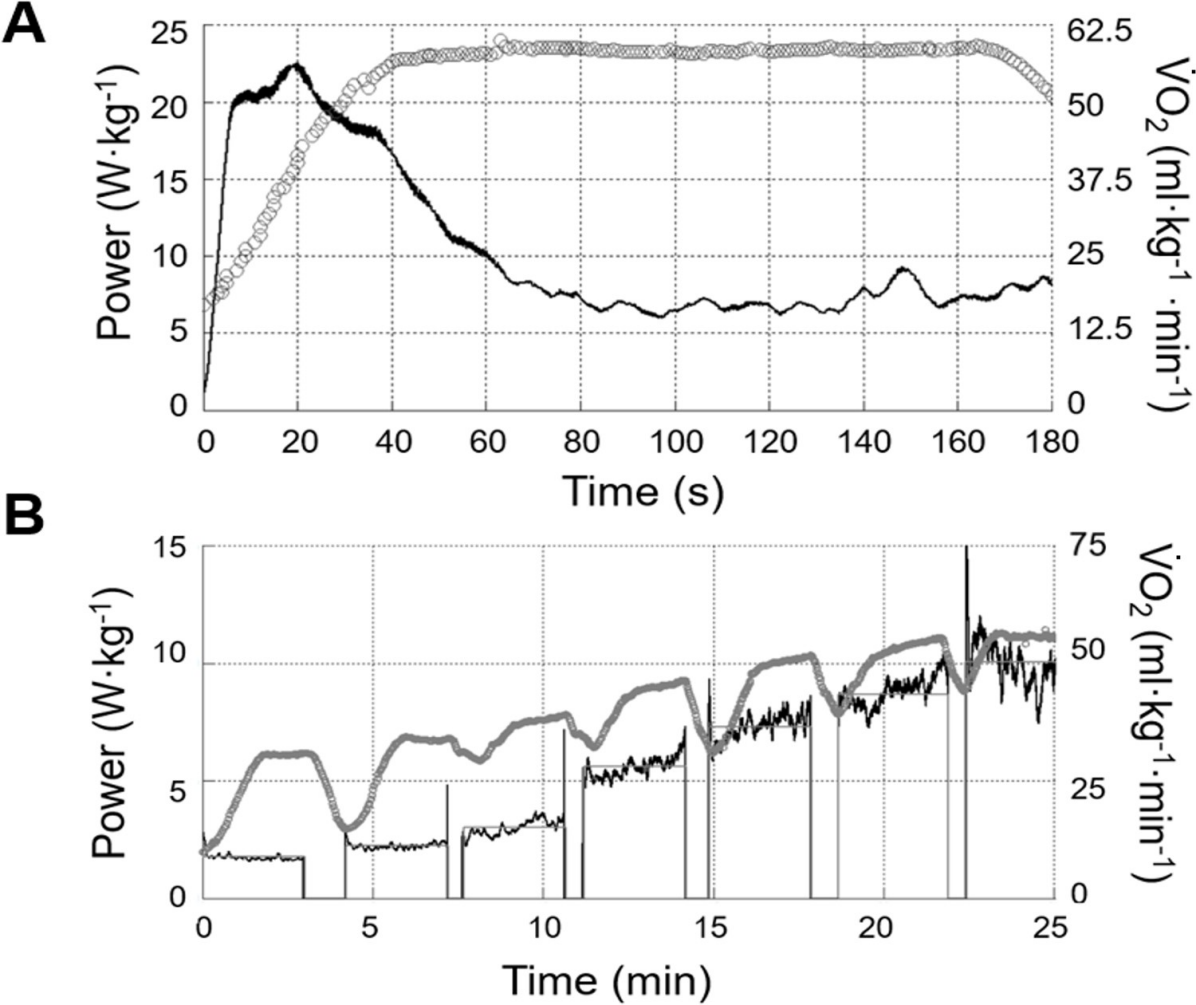
Data example for an AO3 (A) and an incremental intensity test (B). Resultant power is depicted in black, with stage mean work-rate as the straight grey lines. $\dot{V}O_{2}$ is represented by open grey circles and plotted on the right axis.

The incremental power test was conducted using the tethered system on an NMT with online visual feedback. The protocol was composed of 3 minutes stages, with 30 s intervals interspersed for blood sample collection. The initial work rate was 80 W of horizontal power, with step increments of 20 W. The test ended after exhaustion or the incapacity to maintain the intended external load, and the work-rate which $\dot{V}O_{2\max}$ was first attained was considered the $i\dot{V}O_{2\max}$. The CWR with the highest work-rate (147 ± 23% $i\dot{V}O_{2\max}$) and shorter time-to-exhaustion (T_limit_; 166 ± 41 s) was used as a verification bout for $\dot{V}O_{2\max}$ since it consisted of a constant-work-rate effort supra $i\dot{V}O_{2\max}$ [36, 37]. Fig 2 shows an example of one AO3 and one graded exercise test, with resultant power and $\dot{V}O_{2}$ data.

For the first CWR bout, the work rate maintained was set at the peak-power value from the incremental test. For the subsequent CWR, we adjusted the work rate by 20 W of horizontal power, above or below, to maintain all three tests T_limit_ between 2 and 10 minutes. As in the graded exercise test, verbal and visual feedback was provided to control the external load. Inability to maintain external load for more than five consecutive seconds would end the test. Work-rate and T_limit_ were used to calculate classic critical power parameters according to two linear models: Work vs. time (CP1 and Wꞌ1) and Power vs. time^-1^ (CP2 and Wꞌ2). The use of more than one model is intended to confirm the success of the critical power model application in this scenario [38]. We could not calculate the hyperbolic model given the completion of only three CWR efforts.

### 2.4. Equipment and measurements

The NMT employed in this investigation had been used before [41] and was improved with four load cells mounted under the treadmill bed to measure force in the vertical plane [39, 40]. Runners performed all tests tethered by their waists using a non-extensible steel cable in series with another load cell. The resultant force was calculated using the vertical and horizontal plane force measurements, and mechanical power was obtained as the product between velocity and the resultant force. The signal acquisition system (DAQ module, amplifier, load cell, and Hall effect sensor) was set to record data at 1000 Hz. The force signals were filtered using a low-pass, fourth-order Butterworth filter, with cutoff frequency (10 Hz) determined by spectral analysis (fast Fourier transform).

For phase 2 of the study, volunteers ran equipped with a portable gas analyzer (K4b2, Cosmed, Italy) to measure gas exchange at rest, during, and after the test. $\dot{V}O_{2}$, VCO_2,_ and VE were filtered using a rolling average of 30 seconds [41]. For all tests, $\dot{V}O_{2\mathrm{peak}}$ was set as the highest value of the filtered data, to be compared with the incremental test’s verified $\dot{V}O_{2\max}$.

### 2.5. Statistical analyses

Descriptive data are expressed as mean ± standard deviation (SD). The normality of data was tested using the Lilliefors test. T-tests and ANOVA were applied to test for differences between means for two or more parameters, using Scheffé post-hoc test in the latter case, when pertinent. Pearson’s correlation coefficient was used to access the relationship between two parameters not intended to be directly equivalent. All hypothesis testing adopted a significance level of p< 0.05.

Typical error (TE), coefficient of variation (CV%), and ICC were calculated to express error between the criterion and practical parameters, with calculations performed using Will Hopkins spreadsheets [42]. TE was calculated as the standard deviation of the change in scores of raw data divided by square root of 2 and was standardized for measurement units. The same was performed for log-transformed data to back transform it back into the CV%. The ICC used was ICC(3,1), where the "3" refers to the type of ICC where the volunteers are considered as a random effect, but the trials is a fixed effect, and the "1" refers to the use of a single pair of measurements, as opposed to averaged multiple trials [43]. All reliability and agreement parameters are expressed, followed by their respective confidence limits under parenthesis. Thresholds to access the magnitude of standardized TE were <0.1, 0.3, 0.6, 1.0, 2.0, and >2.0 for trivial, small, moderate, large, very large, and impractical. For ICC, thresholds were 0.99, 0.90, 0.75, 0.50, and 0.20, for extremely-high, very-high, high, moderate, and low [42].

Bland-Altman plots of difference vs. mean of the paired scores and limits of agreement were calculated using a custom MatLab (MathWorks Inc, EUA) function. Bias represents the mean difference between pairs of data, while the limits of agreement are the product between the t statistical value for the respective degrees of freedom and the SD of the difference between the pair scores.

## 3. Results

**Fig 3:**
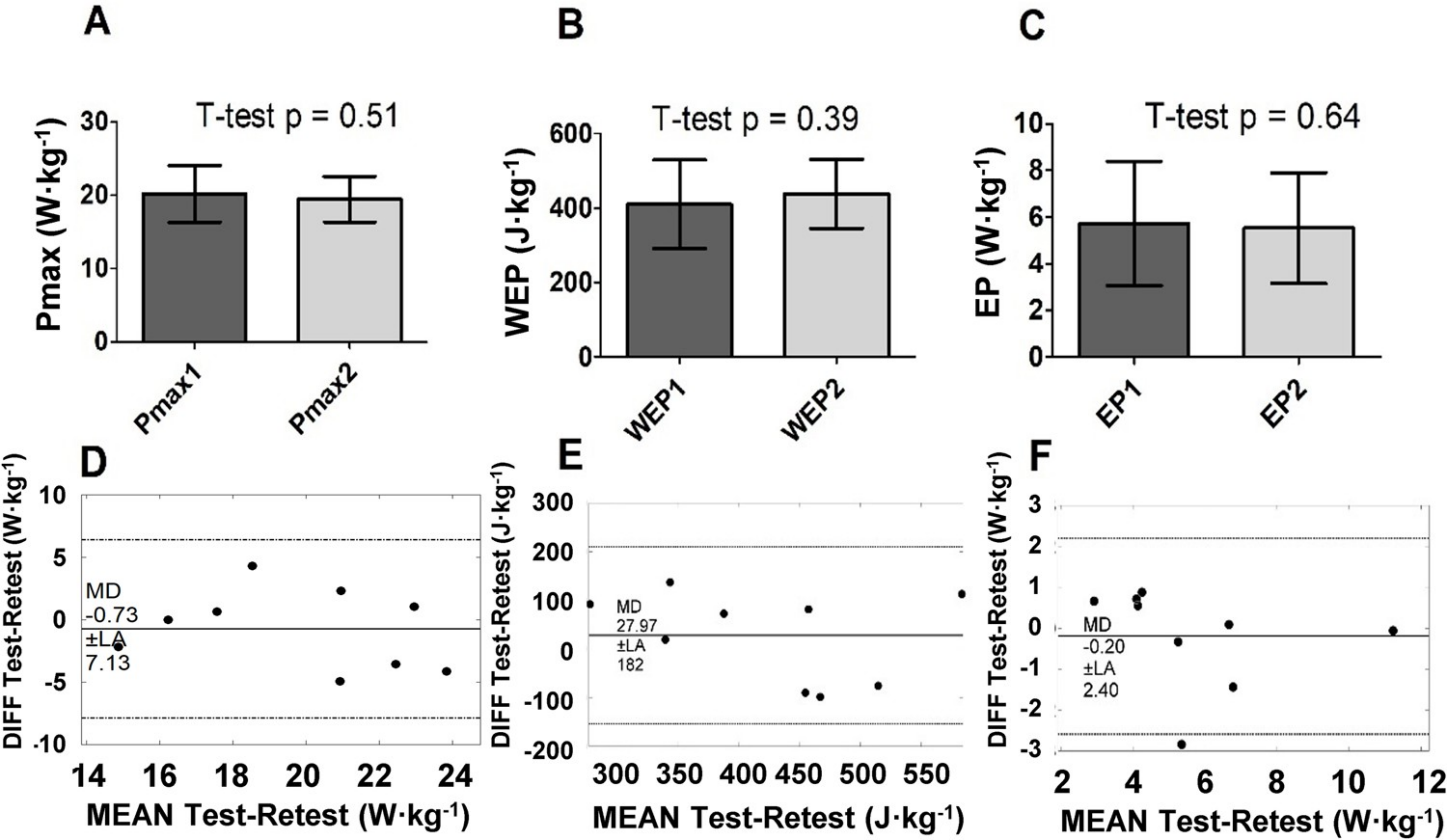
Test-retest comparison of AO3 parameters in mean and SD (Pmax–A; WEP–C; EP–E) and by Bland Altman analysis (Pmax–D; WEP–E; EP–F); (n = 9). Pmax—maximum power; EP–end power; WEP–area above end power.

There were no significant differences between test-retest parameters derived from AO3. Fig 3 shows the descriptive data and respective paired Bland-Altman analysis for Pmax, WEP, and EP, showing generally close to zero bias but wide Limits of Agreement. TE was considered moderate for Pmax and EP, with high ICC for both, but WEP presented lower reliability, with large TE and only moderate ICC (Table 1).

**Table 1:** Reliability analysis and comparison to criterion for power variables derived from AO3 (values in mean and 95%IC).

		Reliability	Agreement to criterion 1	Agreement to criterion 2
Pmax	TE	0.53 (0.38–0.90)	0.70 (0.50–1.20)	-
	CV%	7.8 (5.5–13.7)	9.5 (6.7–16.8)	-
	ICC	0.87 (0.60–0.96)	0.73 (0.30–0.91)	-
EP	TE	0.37 (0.26–0.63)	0.76 (0.54–1.36)	0.72 (0.51–1.30)
	CV%	19.1 (13.4–34.8)	32.8 (22.2–66.6)	32.7 (22.1–66.4)
	ICC	0.86 (0.60–0.96)	0.54 (-0.07–0.85)	0.54 (-0.06–0.85)
WEP	TE	0.78 (0.56–1.73)	1.31 (0.93–2.36)	1.29 (0.91–2.33)
	CV%	17.6 (12.3–31.9)	39.9 (26.7–82.8)	39.7 (26.6–82.4)
	ICC	0.69 (0.22–0.90)	0.32 (-0.33–0.76)	0.28 (-0.36–0.74)

*all reliability data and Pmax agreement were obtained from phase 1, where criterion 1 was Pmax from the 30s all-out (n = 9). Agreement for EP and WEP derived from phase 2 (n = 8), and for those, criterion 1 and 2 were the W/t and the P/t^-1^ models, respectively. TE–typical error; CV%—normalized coefficient of variation; ICC–intraclass correlation coefficient; Pmax–maximum power; EP–end power; WEP–the area above end power.

**Fig 4:**
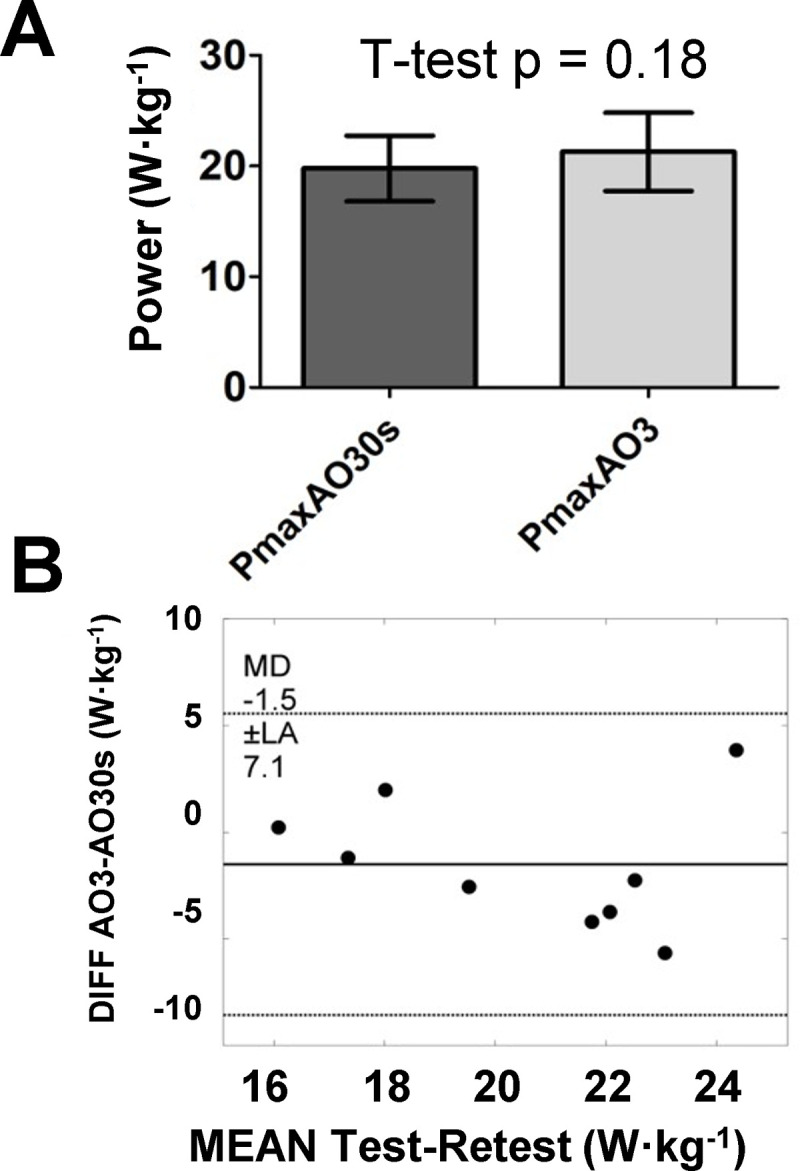
Comparison between Pmax from AO30s and AO3 (A), with the respective Bland-Altman analysis (B); (n = 9). PmaxAO30s –maximum power during the 30-s all-out; PmaxAO3 –maximum power during the AO3.

**Fig 5:**
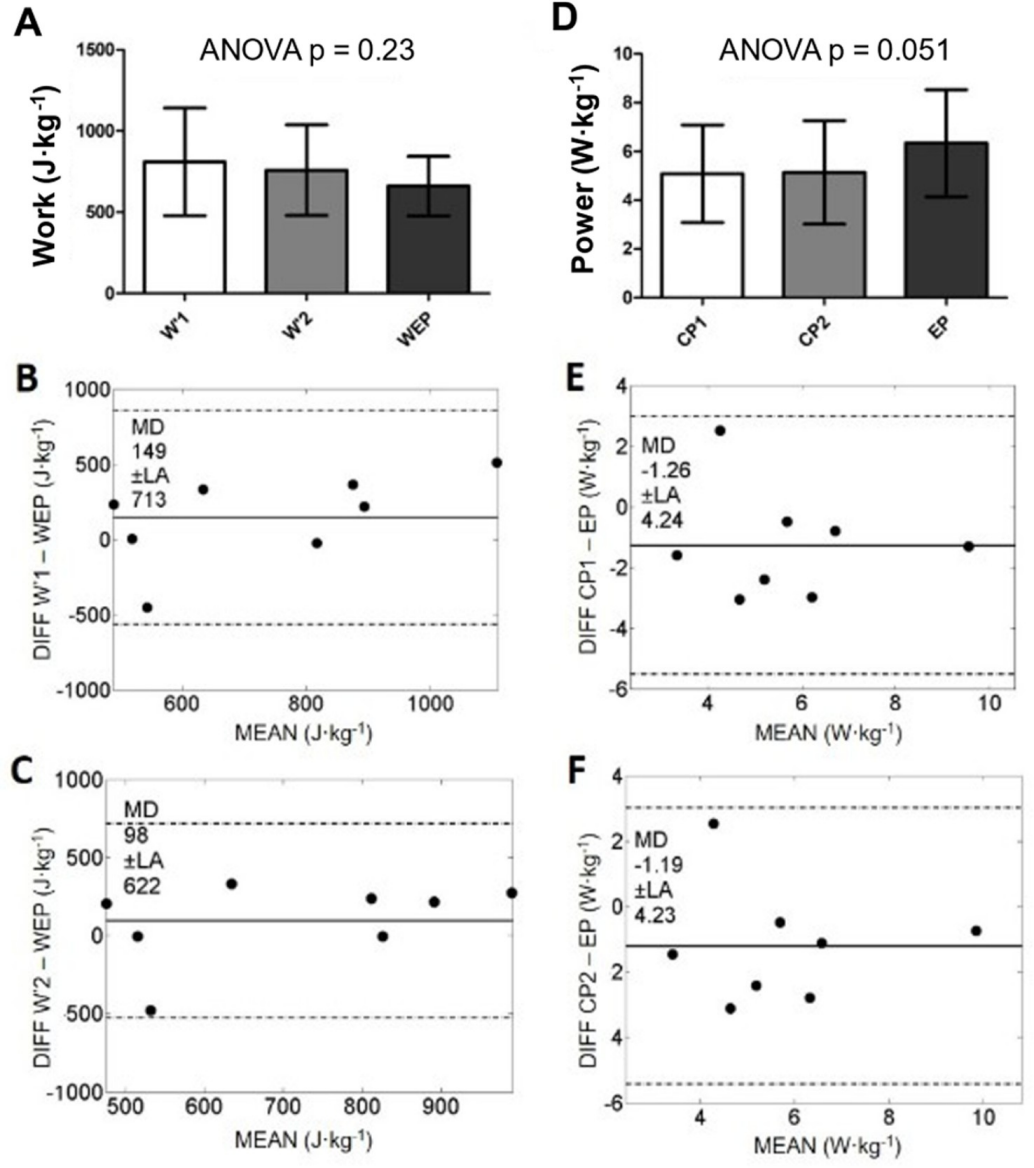
Comparison among WEP and EP derived from AO3 to Wꞌ and CP from the classical critical power models (A and D), and their respective paired Bland-Altman analysis: W1’ vs. WEP (B), Wꞌ2 vs. WEP (C); CP1 vs. EP (E), CP2 vs. EP (F). EP–end power; WEP–area above end power; CP1 –Critical power from model 1; CP2 –Critical power from model 2; Wꞌ1—curvature constant from model 1; Wꞌ2—curvature constant from model 2.

When comparing the Pmax obtained in AO3 to AO30s, no significant differences were present (Fig 4). TE between the two tests continued to be considered moderate for Pmax, but ICC was lower than the reliability comparison (Table 1). Regarding EP (6.34 ± 2.19 W·kg^-1^) and WEP (660 ± 183 J·kg^-1^), there were no significant differences between their respective criteria, despite a tendency of a higher EP (p = 0.051) in comparison to both CP1 (5.08 ± 2.00 W·kg^-1^) and CP2 (5.14 ± 2.12 W·kg^-1^) (Fig 5). The difference can be confirmed based on TE considered large for comparing EP to both CP1 and CP2, with only moderate ICC (Table 1). WEP differences to Wꞌ1 (809 ± 332 J·kg^-1^) or Wꞌ2 (758 ± 278 J·kg^-1^) were even more pronounced, given the very-large TE and low ICC (Table 1). The comparisons to criteria for EP and WEP showed higher errors than these parameters’ test-retest reliability.

Pearson correlations were significant between CP1 and CP2 (r = 0.99; p < 0.001), but no significant correlations were found between CP1 and EP (r = 0.64; p = 0.09) and to CP2 and EP (r = 0.66; p = 0.08). Wꞌ1 and Wꞌ2 were also well correlated (r = 0.98; p < 0.001), but not to EP (r < 0.44).

**Fig 6:**
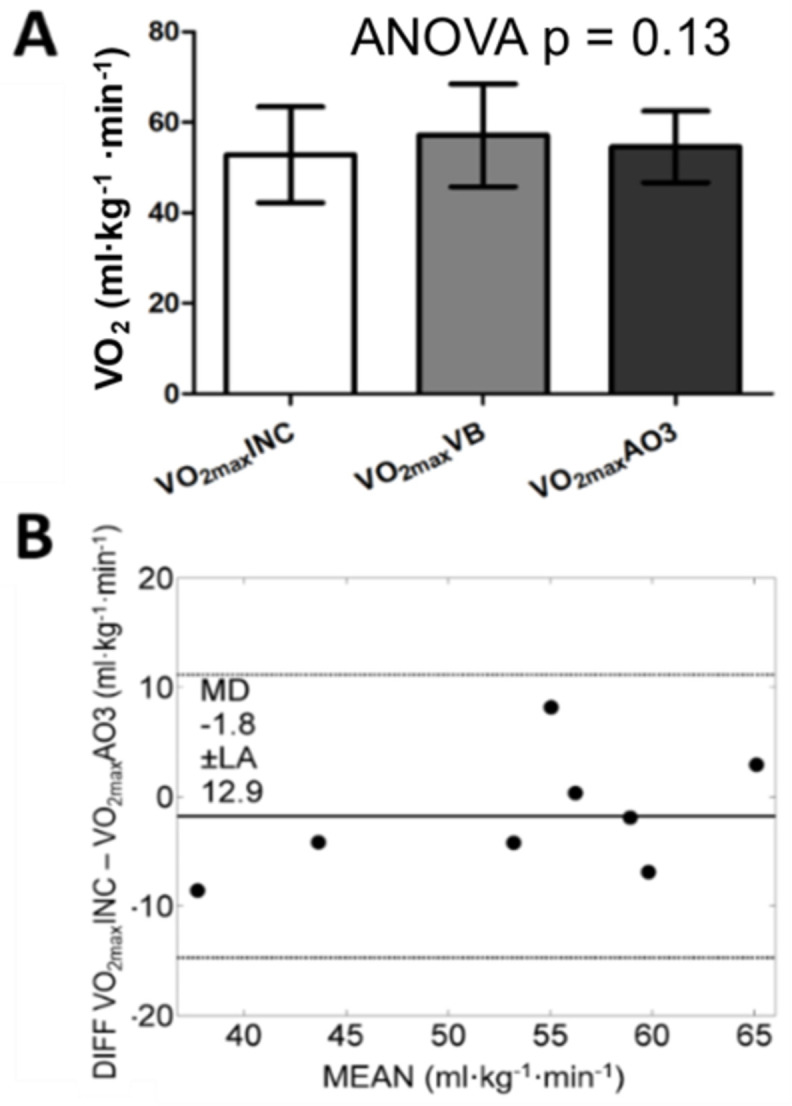
Comparison of $\dot{V}O_{2\max}$ obtained in the graded exercise test (INC), the verification bout (VB) and the AO3 (A), and Bland Altman between INC and AO3 (B).

On the other hand, $\dot{V}O_{2\mathrm{peak}}$ measured during AO3 showed high agreement to the graded exercise test (Fig 6). Peak power output was 6.60 ± 1.36 W·kg^-1^, and the $\dot{V}O_{2\max}$ attainment could be confirmed based on the value from the verification bout. Error between the $\dot{V}O_{2\mathrm{peak}}$ found in the graded exercise test and the verification bout (TE = 0.38; CV% = 6.7; ICC = 0.95) was similar to the comparison with $\dot{V}O_{2\mathrm{peak}}$ in the AO3 (TE = 0.45; CV% = 8.4; ICC = 0.87), with both cases presenting moderate TE and very-high and high ICC.

## 4. Discussion

This study aimed at assessing the reliability and agreement level of parameters obtained during an AO3 (Pmax, EP, WEP, and $\dot{V}O_{2\mathrm{peak}}$) compared to the power-duration relationship, the maximum attained power in shorter exercise, and the graded exercise test during running efforts on a non-motorized treadmill. Overall, Pmax and $\dot{V}O_{2\mathrm{peak}}$ determined during AO3 presented a good agreement with their counterparts. However, sustainable (EP) and non-sustainable (WEP) work capacities were not equivalent to those obtained from the classical critical power model. For instance, the criterion comparison errors were within the reliability variability found for Pmax but more significant for EP and WEP. These results suggest that while running in an instrumented NMT, parameters from the AO3 should not be used as equivalent to the classical CP model.

### 4.1. Reliability of the 3-min all-out test

Test-retest reliability has been monitored for both EP and WEP in cycle ergometry. EP shows better reliability in this exercise scenario with CVs varying 3 to 10% and ICCs higher than 0.89 to 0.99 (1,7). On the other hand, the WEP from AO3 performed on the cycle ergometer returns CV around 21% despite ICCs ranging from 0.76 to 0.98 (7,8), which is closer to those presented here. AO3 reliability was also measured for the rowing ergometer, returning test-retest CV of 9% for EP and ICCs of 0.78 for EP and 0.62 for WEP [14].

With few studies focused on the AO3 application for running and controlling external load only based on velocity (23,24), Gama et al. [33] were the first to study the AO3 reliability on a non-motorized treadmill measuring mechanical power. Based on the data from Gama et al. [33], reliability is improved when measurements are based on power rather than on force or velocity. Our results confirm the intra-subject reliability of Pmax, WEP, and EP for running on a non-motorized treadmill. This assumption is based on ICC and TE analysis. However, when considering the magnitude of the limits of agreement and CV, one should consider the individual variability for these parameters when studying training adaptations. Despite our data showing high ICC and only moderate TE for EP, a CV of 19% should be expected, higher than those from other exercise scenarios.

### 4.2. Agreement between parameters

Despite experimental data indicating proper reliability [8] and a good agreement for EP compared to CP [44] on the cycle ergometer, there are also experimental pieces of evidence to challenge EP equivalence to CP. Among them, it is worth noting a poor agreement between EP and the conventional CP [9, 22]. As well, EP is not considered a sustainable work rate for elite cyclists (T_limit_ = 14.79 ± 8.38 minutes) [45] or moderately trained men and women (T_limit_ = 12.5 ± 6.5 minutes) [46]. In the current study, EP presented a high typical error compared to CP for both linear models and was not significantly correlated to any of them. Further, a high bias for the comparison between WEP and Wꞌ has been found elsewhere [22, 24, 44] and in the present study, despite strong evidence suggesting a physiological meaning for WEP as representative of metabolic pathways independent of oxygen [47].

Gama et al. [34] showed no significant difference, bias close to zero but a large limits of agreement between EP and CP derived from three different classical CP models. On the other hand, WEP agreed much less with Wꞌ from the models applied, presenting significant differences, close to zero ICCs, and large bias and error.

The present study furthers the work of AO3 application on an NMT, presenting a setup where the external load can be controlled by visual feedback from the performed power. This setup enables the increase of the work rate to determine traditional CP based on the force or velocity applied voluntarily by the runner, as occurs in a free-running scenario. The application presented by Gama and colleagues increased the intensity by adding resistance from elastic chords attached to the runner’s waist. A different resistance characteristic may explain a slightly better agreement between classical CP and EP in Gama et al. [34] than the data provided here. With cycle ergometry, for example, the imposed resistance may influence EP and WEP’s agreement with the classical CP parameters [27, 48]. Future studies may focus on the force-velocity or power-force relationships with the NMT to identify if the resistance settings may enhance EP and WEP reliability and validity for running exercise, which may improve the agreement of AO3 parameters with the classical CP parameters.

Unlike what may be expected, the Pmax attained during AO3 showed good agreement with the parameter from AO30s. Previous investigations have shown lower peak power output with longer all-out effort durations [49, 50]. This feature has been attributed to a psychological factor rather than bioenergetic. Once aware of the long effort’s physiological consequences, the individual may approach the effort with care, thus failing to activate the neuromuscular system [50]. Previous data, however, has shown the effect of submaximal work-rate to be less pronounced when comparing all-out lasting 20 to 30 seconds [49]. Data presented in our study show no significant decrease of Pmax between the thirty-second and the three-minute all-out efforts, suggesting that this effect of exercise duration on Pmax reduction may level off with the longer exercise duration.

Regarding $\dot{V}O_{2\mathrm{peak}}$, the values obtained from the AO3 were not different from those obtained from the traditional graded exercise test. This finding confirms that even with the setup used in our study, the AO3 is capable of eliciting $\dot{V}O_{2\max}$. Previous research has shown no difference comparing $\dot{V}O_{2\max}$ from a ramp test on a motorized treadmill with peak $\dot{V}O_{2}$ attained during AO3 performed on a 400-m track. However, considering a moderate to large effect size between these two parameters, Sperlich et al. [18] argue that $\dot{V}O_{2\max}$ was not attained during AO3. On the other hand, Kramer et al. [51] confirmed $\dot{V}O_{2\max}$ attainment on shuttle running. Differences in $\dot{V}O_{2}$ data treatment may explain the conflicting results since Kramer et al. [51] used 1-second averages while Sperlich et al. [18] used a fixed window of 30-second averages for the same. The current investigation used a rolling average and found comparable values for $\dot{V}O_{2\mathrm{peak}}$ from the graded exercise test, the verification bout, and the AO3, with moderate TE and high ICC. Thus, the $\dot{V}O_{2}$ data filtering procedures in previous studies may be responsible for controversial results about the $\dot{V}O_{2\mathrm{peak}}$ attained during an AO3. In the current study, using a rolling average on $\dot{V}O_{2}$ data from the AO3, we were able to attain a $\dot{V}O_{2\mathrm{peak}}$ comparable to the verified $\dot{V}O_{2\max}$ from an incremental test.

### 4.3. Study’s limitations and future perspectives

Among the study’s limitations is the duration of the CWR. Although CWR lasting between 2 and 10 minutes have been classically used, it was proposed that more prolonged CWR could improve agreement between CP models [52]. A combination of the CWR durations like the ones used here may overestimate CP while underestimating Wꞌ [52]. Considering the present results, CP tended to be lower than EP, while Wꞌ was higher than WEP. So, enhancing the CWR durations could only widen the difference between them, which does not change the conclusions drawn here.

Finally, most of the previous results on AO3 parameters have been performed in cycle ergometry, where power exertion can be better controlled. However, the data presented here are relevant since multiple studies are already applying the AO3 while testing for training adaptations in running exercises. An instrumented NMT was chosen to present power output in both vertical and horizontal orientations, minimizing this issue. Slight differences in the running pattern could be noted between running on the NMT and overground.

Future studies may attempt to test AO3 parameters on an NMT with differences in the applied resistance to try to find better results on validity and reliability. Still, confirmation rides should be performed [53] to verify time to exhaustion and physiological responses at EP and CP, and access if the differences shown here are physiologically meaningful.

In conclusion, no significant differences were found between EP and WEP to their criteria. However, considering the large limits of agreement, low intra subject consistency and high CV, EP and WEP should not be used interchangeably to CP and Wꞌ using a NMT as described in the setup of this study. On the other hand, EP, WEP, and Pmax were test-retest reliable, $\dot{V}O_{2\mathrm{peak}}$ was considered equivalent to $\dot{V}O_{2\max}$, and Pmax agreed with the criteria established.

## Supporting information

S1 Data Raw data for both experiments described in the manuscript. The spreadsheet labeled as Experiment 1 contains the teste-retest data for AO3 and the data for AO30s. Also, it is possible to find AO3 data, both models critical power data and VO2max data in the spreadsheet named Experiment 2.
(XLSX)
