# Correction: Comparison of parameters derived from a three-minute all-out test with classical benchmarks for running exercise

**DOI:** 10.1371/journal.pone.0356340

**Published:** 2026-08-18

**Authors:** Filipe A. B. Sousa, Fúlvia B. Manchado-Gobatto, Natália A. Rodrigues, Claudio A. Gobatto

Following publication of this article [1], concerns were raised regarding Fig 6A and Table C1 in the Supporting Information file S1 Data. The *PLOS One* Editors sought input about these issues from independent members of the Editorial Board.

As the result of a data entry error, a specific value presented in the Supporting Information and associated values in the main article and figures are incorrect.

This Correction addresses the following specific errors:

- In tab Experiment 2 in S1 Data, the value of VO2maxVB for Subject #4 is incorrect.
- As a result of the above error, the ANOVA analysis displayed in Fig 6A of [1] is incorrect. The analysis has been rerun and the error does not change the result substantially (*p* = 0.106 instead of the original *p* = 0.130). An updated, correct version of Fig 6 is provided with this notice.
- The error comparison between VO2maxINC and VO2maxVB was rerun. In the Results section, there is an error in the third sentence of the fourth paragraph. The correct sentence is: “Error between the VO2peak found in the graded exercise test and the verification bout (TE = 0.41; CV% = 7.2; ICC = 0.94) was similar to the comparison with VO2peak in the AO3 (TE = 0.45; CV% = 8.4; ICC = 0.87), with both cases presenting moderate TE and very-high and high ICC.” The authors indicated that the slight changes in the statistical values do not change the interpretation of the results nor affect the conclusions of the paper, and that no other analyses were affected by this mistake. A member of the Editorial Board reviewed this and agreed that the study's conclusions remain supported.

The authors have provided the corrected data as Supporting Information file S1 Data. With this Correction, all relevant data are now provided. The authors apologize for the errors in the published article.

Additionally, a member of the Editorial Board raised the issue of a potentially limited sample size and consequently the authors performed a post-hoc statistical power analysis for the ANOVA in Fig 6A. The G*Power software was used. Considering the high correlation between conditions (*r* = 0.86), the high effect size obtained by *η*^2^ (0.61), and even using the most conservative nonsphericity correction (0.5), the authors obtained a statistical power of 0.99 for the ANOVA comparison between VO2maxINC, VO2maxVB and VO2max AO3.

**Fig 6:**
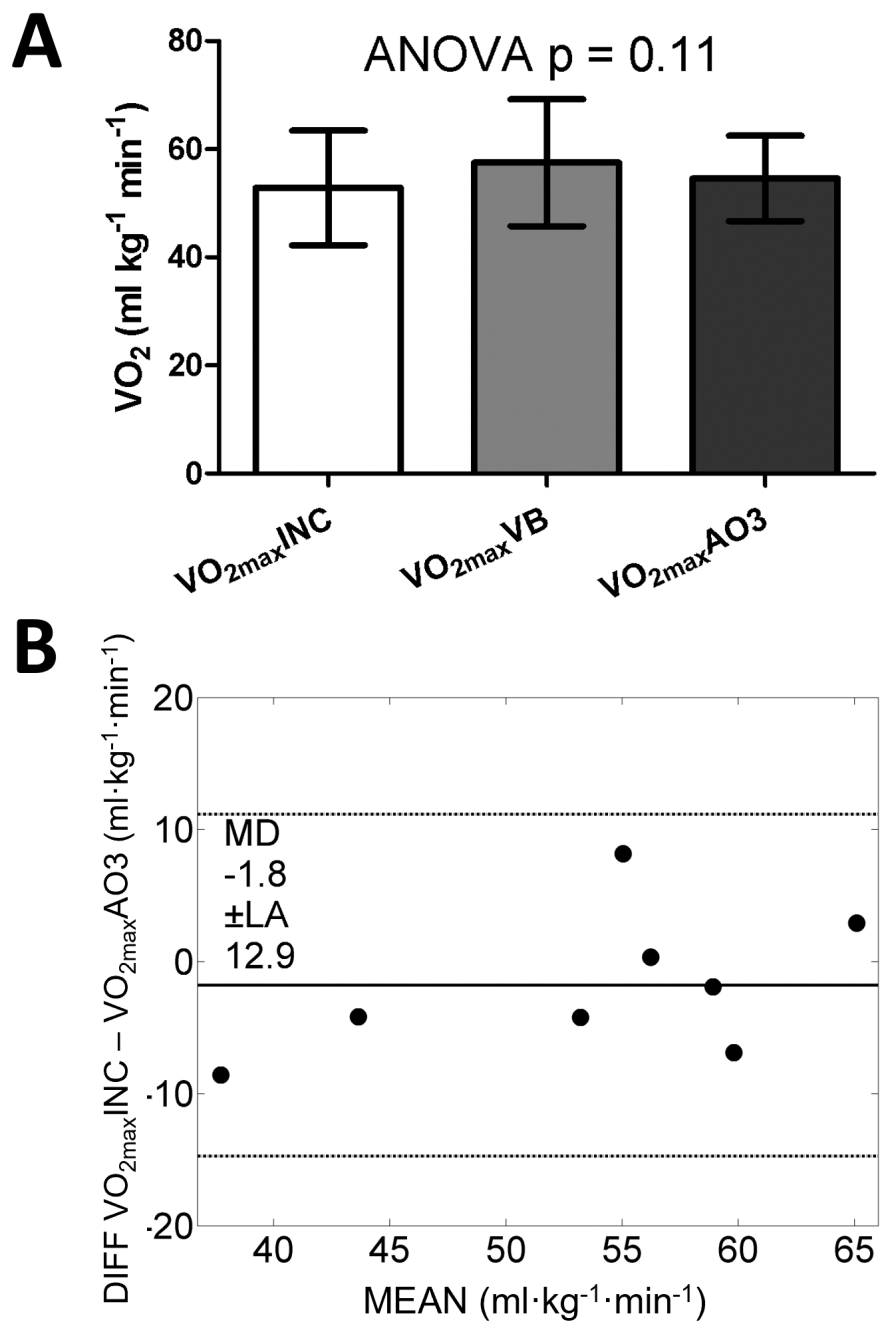
Comparison of VO2max obtained in the graded exercise test (INC), the verification bout (VB) and the AO3 (A), and Bland Altman between INC and AO3 (B).

## Supporting information

S1 Data Raw data for both experiments described in the manuscript. The spreadsheet labeled as Experiment 1 contains the test-retest data for AO3 and the data for AO30s. Also, it is possible to find AO3 data, both models critical power data and VO2max data in the spreadsheet named Experiment 2.
(XLSX)
